# Measuring glucose cerebral metabolism in the healthy mouse using hyperpolarized ^13^C magnetic resonance

**DOI:** 10.1038/s41598-017-12086-z

**Published:** 2017-09-15

**Authors:** Mor Mishkovsky, Brian Anderson, Magnus Karlsson, Mathilde H. Lerche, A. Dean Sherry, Rolf Gruetter, Zoltan Kovacs, Arnaud Comment

**Affiliations:** 1 Laboratory for Functional and Metabolic Imaging, Ecole Polytechnique Fédérale de Lausanne (EPFL), CH-1015 Lausanne, Switzerland; 2 Institute of Physics of Biological Systems, Ecole Polytechnique Fédérale de Lausanne, CH-1015 Lausanne, Switzerland; 3 Advanced Imaging Research Center, University of Texas Southwestern Medical Center, Dallas, Texas 75390-8568 USA; 4 Albeda Research ApS, Ole Maaløes vej 3, 2200 Copenhagen, Denmark; 5 Technical university of Denmark, Department of Electrical Engineering, 2800 Kgs, Lyngby, Denmark; 6 Department of Radiology, Université de Lausanne, CH-1015 Lausanne, Switzerland; 7 Department of Radiology, Geneva University Hospital and Faculty of Medicine, University of Geneva, CH-1205 Genève, Switzerland; 8 General Electric Healthcare, Chalfont St Giles, Buckinghamshire, HP8 4SP United Kingdom

## Abstract

The mammalian brain relies primarily on glucose as a fuel to meet its high metabolic demand. Among the various techniques used to study cerebral metabolism, ^13^C magnetic resonance spectroscopy (MRS) allows following the fate of ^13^C-enriched substrates through metabolic pathways. We herein demonstrate that it is possible to measure cerebral glucose metabolism *in vivo* with sub-second time resolution using hyperpolarized ^13^C MRS. In particular, the dynamic ^13^C-labeling of pyruvate and lactate formed from ^13^C-glucose was observed in real time. An ad-hoc synthesis to produce [2,3,4,6,6-^2^H_5_, 3,4-^13^C_2_]-D-glucose was developed to improve the ^13^C signal-to-noise ratio as compared to experiments performed following [U-^2^H_7_, U-^13^C]-D-glucose injections. The main advantage of only labeling C3 and C4 positions is the absence of ^13^C-^13^C coupling in all downstream metabolic products after glucose is split into 3-carbon intermediates by aldolase. This unique method allows direct detection of glycolysis *in vivo* in the healthy brain in a noninvasive manner.

## Introduction

Dynamic *in vivo* ^13^C MRS combined with the injection of ^13^C-enriched substrates is a powerful method for studying cerebral intermediary metabolism^1^. It is well established that, although the energy requirement of the brain can be satisfied by the oxidation of other substrates such as ketone bodies, lactate and fatty acids, glucose is the main cerebral metabolic fuel^2^. The improved sensitivity and resolution of high-magnetic field ^13^C MRS has increased the reliability of ^13^C-enrichment time evolution measurements so that metabolic fluxes can now be determined accurately. The temporal resolution currently achievable in the rodent brain upon infusion of [1,6-^13^C_2_]-D-glucose is around 5 min for detection of ^13^C labeling in the aliphatic carbons of glutamate, glutamine and aspartate, and about 20 min for detection of less concentrated metabolites such as γ-aminobutyrate, alanine and lactate^3^. Other important intermediary metabolites present in low concentration such as pyruvate can simply not be detected in the rodent brain by conventional thermally polarized ^13^C MRS. Over the past few decades, multiple technological advances have been implemented to overcome the inherent low sensitivity of nuclear magnetic resonance (NMR) and MRS^4^. This lack of sensitivity comes from the fact that the NMR/MRS signal is directly proportional to the nuclear spin polarization *P*_n_ defined as the relative difference between the populations of the different nuclear spin quantum states. This polarization is inherently small at room or body temperature even in a magnetic field as high as 9.4T where *P*_n_ < 8 × 10^−6^ for ^13^C spins at 37 °C. To enable detection of less concentrated metabolites *in vivo*, a tremendous ^13^C signal enhancement in the biomolecules of interest can be achieved using so-called “hyperpolarization” techniques. The development of hyperpolarization, in particular dissolution dynamic nuclear polarization (DNP) which can increase the room-temperature ^13^C polarization of molecules in solution by several orders of magnitude^5^, leads to the possibility of following uptake and metabolism *in vivo* in real time^6^. To obtain large polarization enhancements by DNP, it is necessary to prepare frozen glassy solutions containing a labeled substrate at high concentration and a few tens of mM of suitable paramagnetic centers as polarizing agents. The frozen samples are placed in a polarizer, an instrument operating at moderately large magnetic field (~3–7 T) and low temperature (~1 K).

To date, only a restricted number of hyperpolarized ^13^C MRS brain studies have been performed^7^, mostly using pyruvate as substrate. Three metabolic products, lactate, alanine and bicarbonate^8,9^, were observed following the injection of [1-^13^C]pyruvate while glutamate and citrate were detected using hyperpolarized [2-^13^C] pyruvate^10^. Following the infusion of hyperpolarized [1-^13^C] acetate, it was also reported that the TCA cycle intermediate 2-oxoglutarate can be observed in the rat brain^11^. Unlike the carbonyl carbon in pyruvate, glucose carbons have very short longitudinal relaxation time (T_1_) (~1–2 s) because of the dipolar-dipolar relaxation of the ^13^C nuclear spins by the protons present in the molecule. In the perdeuterated [U-^2^H_7_, U-^13^C_6_]-D-glucose, the intramolecular dipolar-dipolar ^13^C-^1^H relaxation is eliminated, which extends the T_1_s of the glucose carbons to ~10 s. It was demonstrated that [U-^2^H_7_, U-^13^C_6_]-D-glucose may be used as a hyperpolarized ^13^C MRS probe to observe glycolytic reactions^12–15^. Hyperpolarized [U-^2^H_7_, U-^13^C_6_]-D-glucose and fructose as nutrients allowed collecting real-time metabolic data from the appearance of downstream metabolites in glycolysis and central carbon metabolism in Escherichia coli and Saccharomyces cerevisiae cells. Perdeuterated [U-^13^C_6_]-D-glucose was also used to successfully probe glycolysis in human T47D breast cancer cells.

It was recently demonstrated that glycolysis in EL4 tumors in mice can be imaged *in vivo* with [U-^2^H_7_, U-^13^C_6_]-D-glucose^16^. While the C_1_ lactate signal was clearly observable as a doublet, these data also highlighted the major drawback of perdeuterated [U-^13^C_6_]-D-glucose as a hyperpolarized glycolytic probe: the splitting of the lactate signal on top of the short ^13^C T_1_ further lowers the signal-to-noise (SNR) ratio. In this particular article, it was suggested that developing a 3-, 4-, or 3,4-^13^C-labeled perdeuterated glucose would lengthen the longitudinal relaxation time of the ^13^C nuclei by eliminating the ^13^C-^13^C homonuclear dipolar relaxation^16^. In addition, this labeling strategy will also metabolize to singly [1-^13^C]-labeled lactate, improving the detection limits of lactate. The aim of the present study was to show that cerebral glucose metabolism can be measured *in vivo* using hyperpolarized ^13^C MRS. We demonstrated that several metabolites can be detected in the healthy mouse brain following the i.v. injection of hyperpolarized ^13^C-glucose. We also developed a convenient synthesis to produce [2,3,4,6,6-^2^H_5_, 3,4-^13^C_2_]-D-glucose on a multigram scale and demonstrate that it has improved characteristics as a hyperpolarization probe for detecting real-time glucose metabolism.

## Results

### Synthesis of [2,3,4,6,6-^2^H_5_, 3,4-^13^C_2_]glucose

**Figure 1:**
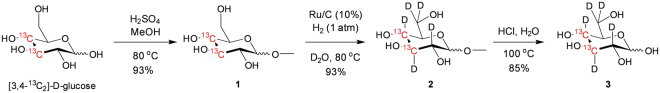
Synthesis of [2,3,4,6,6-^2^H_5_, 3,4-^13^C_2_]glucose.

Due to the cost of [3,4-^13^C_2_]-D-glucose starting material, our goal was to develop a simple, high yield route to prepare deuterated glucose. The established methods for the deuteration of carbohydrates rely on the replacement of covalently bound hydrogens with deuterium using D_2_O and a transition metal catalyst such as Raney Ni or Ru/C^17–21^. After careful consideration of the literature data and some preliminary experimentation, we adopted the Ru/C method largely because the deuterated Raney Ni catalyst required long reaction times, which lead to significant isomerizations and formation of other side products^19,22^. The synthesis of [2,3,4,6,6-^2^H_5_, 3,4-^13^C_2_]-D-glucose is outlined in Fig. 1. It is known that prolonged direct treatment of glucose with Ru/C, H_2_ and D_2_O leads to complete decomposition^21^. Consequently, we first converted [3,4-^13^C_2_]-D-glucose to the methyl glucoside (**1**) under standard conditions by stirring the compound in sulfuric acid in methanol at reflux overnight. The resulting methyl glucoside was then dissolved in D_2_O, the Ru/C catalyst was added, and the reaction mixture was stirred under an atmosphere of hydrogen at 80 °C. This resulted in incomplete deuteration largely at the C3 position. Simple removal of the used Ru/C by filtration and adding fresh catalyst proved to be an efficient way of increasing the deuteration. This iteration was repeated until the desired percent deuteration was achieved (>95% deuteration required three repetitions). The final step of the synthesis was the hydrolysis of the glycosidic bond by refluxing the deuterated product in 1 M HCl. The overall yield of the desired final product was about 75% starting from [3,4-^13^C_2_]-D-glucose.

### Liquid-state polarization

Using commercially available DNP hardware, the back calculated liquid-state ^13^C polarization at the time of dissolution was about 30% for αC3 and αC4 for both [2,3,4,6,6-^2^H_5_, 3,4-^13^C_2_]-D-glucose and the perdeuterated, uniformly labeled derivative, while the αC1 ^13^C polarization in the latter compound was measured to be 13% (see ref.^23^, Tables S1 and S2, as well as Fig. S1). The liquid-state T_1_ values for both compounds were measured at 9.4T and room temperature after dissolution with water or D_2_O. These T_1_s were determined from the fitting of the hyperpolarized magnetization decay curves. As seen from the values displayed in Table 1, the apparent T_1_s of the 3,4-^13^C_2_-labeled and the uniformly labeled derivatives do not differ significantly. Deuteration of the solvent increased the T_1_s by approximately 20%, in agreement with previously reported results in which it was shown that dipole-dipole relaxation is the major relaxation mechanism for ^13^C spins in carbohydrates^24^.

**Table 1:** T_1_ values of C3 and C4 carbons of [2,3,4,6,6-^2^H_5_, 3,4-^13^C_2_]-D-glucose and [U-^2^H_7_, U-^13^C_6_]-D-glucose in H_2_O and D_2_O at 9.4T.

T_1_ (s)^a^ [2,3,4,6,6-^2^H_5_, 3,4-^13^C_2_]-D-glucose				T_1_ (s)^a^ [U-^2^H_7_, U-^13^C_6_]-D-glucose			
C3		C4		C3		C4	
H_2_O	D_2_O	H_2_O	D_2_O	H_2_O	D_2_O	H_2_O	D_2_O
12.0 ± 0.2	13.9 ± 0.4	11.0 ± 0.4	12.1 ± 0.3	10.4 ± 0.3	12.2 ± 0.3	10.9 ± 0.3	12.5 ± 0.3

The T_1_ values were determined from the fitting of the hyperpolarized magnetization decay curves taking into account the decay by T_1_ relaxation and RF pulsing. The data were collected using 5 degree pulse angle and repetition time of 5 s. The samples did not contain Gd^3+^.

^a^An average of three runs.

The liquid-state polarization of hyperpolarized [U-^2^H_7_, U-^13^C_6_]-D-glucose was measured inside a custom-designed injection pump following DNP at 7T/1K, dissolution in D_2_O and transfer into the bore of a 9.4T MR scanner, using a previously described method^25,26^. The polarization was determined by comparing the hyperpolarized ^13^C-glucose signal and its corresponding thermally polarized signal (see example in Fig. S2). The average ^13^C polarization values are presented in Table 2. These correspond to the polarization levels at the time of the animal intravenous (i.v.) injection.

**Table 2:** Liquid-state polarization measured inside the infusion pump (n = 3).

Carbon position (α + β)	Polarization %
C_1_	18.9 ± 1.1
C_2–5_	22.2 ± 2.1
C_6_	18.5 ± 2.2

### *In vivo*^13^C MRS

**Figure 2:**
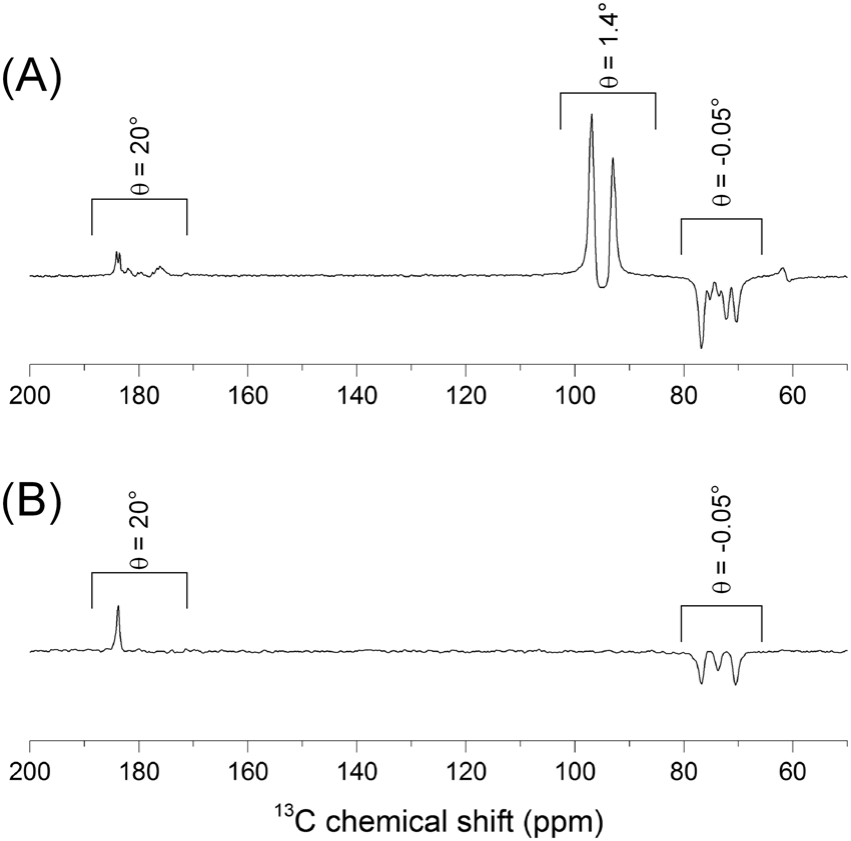
Effective flip angle during the acquisition of hyperpolarized glucose metabolism *in vivo*. Summed 13C spectra recorded in the mouse head following the injection of hyperpolarized [|U-^2^H_7_, U-^13^C_6_]-D-glucose (**A**) and [2,3,4,6,6-^2^H_5_, 3,4-^13^C_2_]-D-glucose (**B**). While the flip angle on the metabolites carboxyl carbon resonances is 20° its effect on the glucose resonance is minimal. The effective flip angle on glucose resonances was confirmed in phantom measurements.

To optimize signal-to-noise ratio (SNR) of the expected glucose metabolites in the carboxyl region of the ^13^C spectra, in particular [1-^13^C] lactate and [1-^13^C] pyruvate, we applied an RF excitation pulse designed to excite the glucose resonances by a minute flip angle, while exciting the region of interest with a large flip angle (20°) (See Fig. 2A and B). The latter was determined using the following considerations: taking into account the published *in vivo* ^13^C T_1_ of perdeuterated [U-^13^C_6_] glucose of 9 s^16^, the maximum SNR that can be obtained after summing all spectra recorded with an achievable repetition time (greater or equal to 500 ms) corresponds to a flip angle of 20° (see Fig. 8 in ref.^27^). This strategy allowed following build-up of glucose metabolites for more than 30 s.

**Figure 3:**
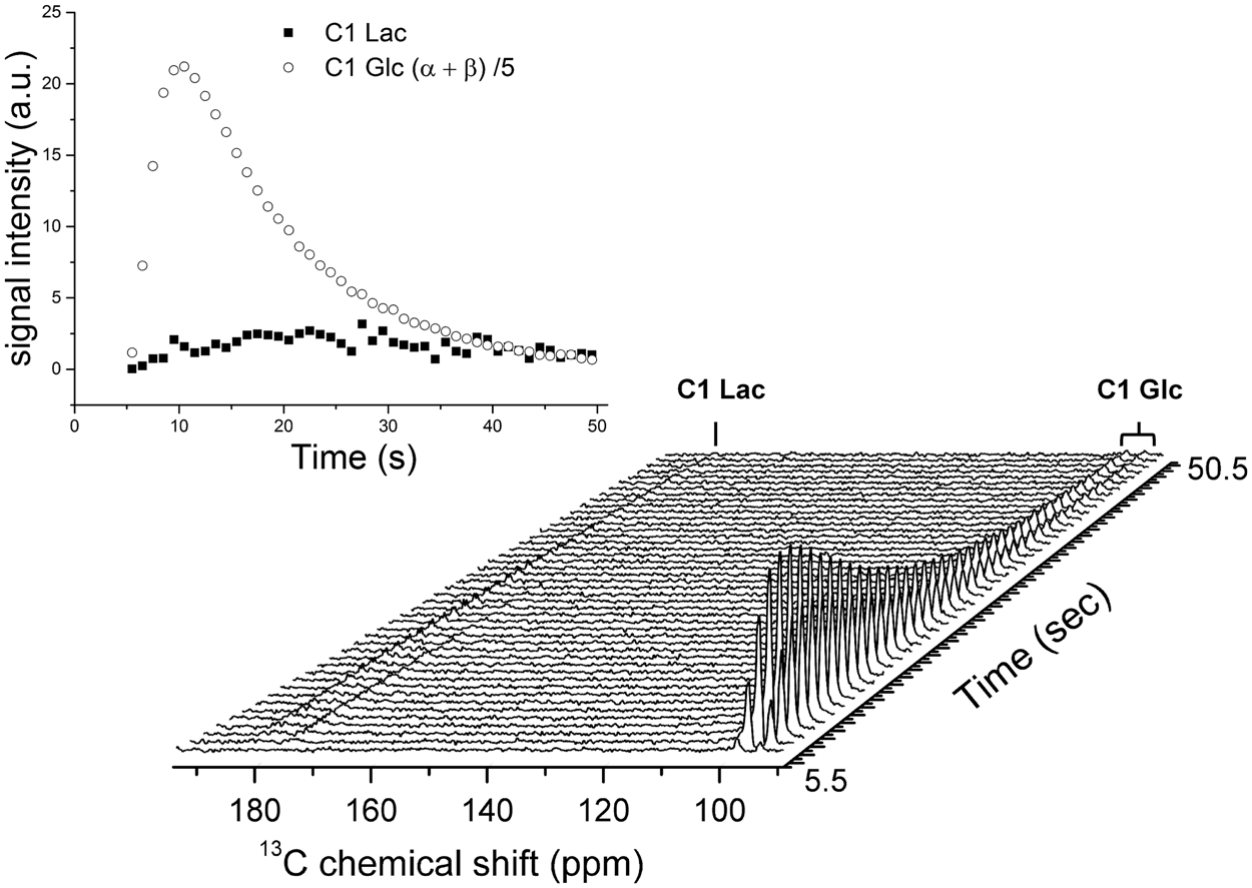
*In vivo* time evolution of [U-^2^H_7_, U-^13^C_6_]-D-glucose and its metabolic product [^13^C_3_]lactate in the mouse head. A 20° flip angle pulse was applied every 0.5 s on the carboxyl region of the spectrum while the flip angle was measured to be 1.4° on the substrate region. To improve the SNR every two spectra were added leading to actual time resolution of 1 s. Glucose C_1_ peaks appear at 96.8 ppm (C_1_-β) and 93 ppm (C_1_-α), lactate is observed at 183.5 ppm and can be identified 10.5 s after the infusion (bottom). The corresponding time course (inset) of the spectral data that was quantified using AMARES while fitting the lactate doublet to two separated peaks.

**Figure 4:**
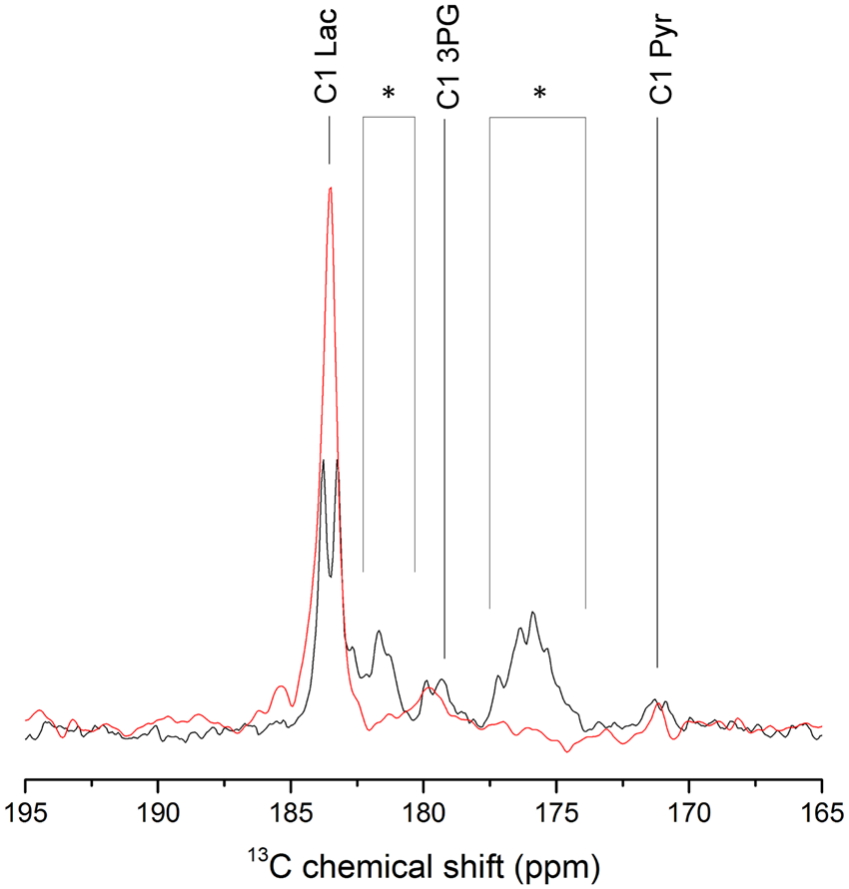
Comparison between the ^13^C signals obtained in the mouse head following the injection of either hyperpolarized [U-^2^H_7_, U-^13^C_6_]-D-glucose or hyperpolarized [2,3,4,6,6-^2^H_5_, 3,4-^13^C_2_]-D-glucose. Zoomed carboxyl region in the summed spectra acquired after infusion of hyperpolarized [U-^2^H_7_, U-^13^C_2_]-D-glucose (black) or hyperpolarized [2,3,4,6,6-^2^H_5_, 3,4-^13^C_2_]-D-glucose (red). The glycolytic intermediates 3PG (179.8 ppm) and pyruvate (171.1 ppm) can be identified in addition to the lactate peak at 183.5 ppm. The resonances marked by (*) are unknown impurities. The ^13^C-^13^C coupling pattern collapses to a single peak when replacing [U-^2^H_7_, U-^13^C_2_]-D-glucose by [2,3,4,6,6-^2^H_5_, 3,4-^13^C_2_]-D-glucose.

Subsequent to the injection of perdeuterated [U-^13^C_6_]-D-glucose, the formation of a doublet peak assigned to the lactate resonance could be readily detected at 183.5 ppm with 50 Hz ^13^C-^13^C coupling (Fig. 3). In the summed spectrum, two additional doublet peaks were detected: one centered at 171.1 ppm with 43 Hz ^13^C-^13^C coupling corresponding to the pyruvate C1 resonance and an additional peak centered at around 179.8 ppm with 56 Hz ^13^C-^13^C coupling, the latter could either be assigned to the glycolytic intermediate [1-^13^C] 3-phosphoglycerate ([1-^13^C] 3PG) or to the pentose phosphate pathway intermediate [1-^13^C] 6-phosphogluconate ([1-^13^C] 6PG) (Fig. 4).

**Figure 5:**
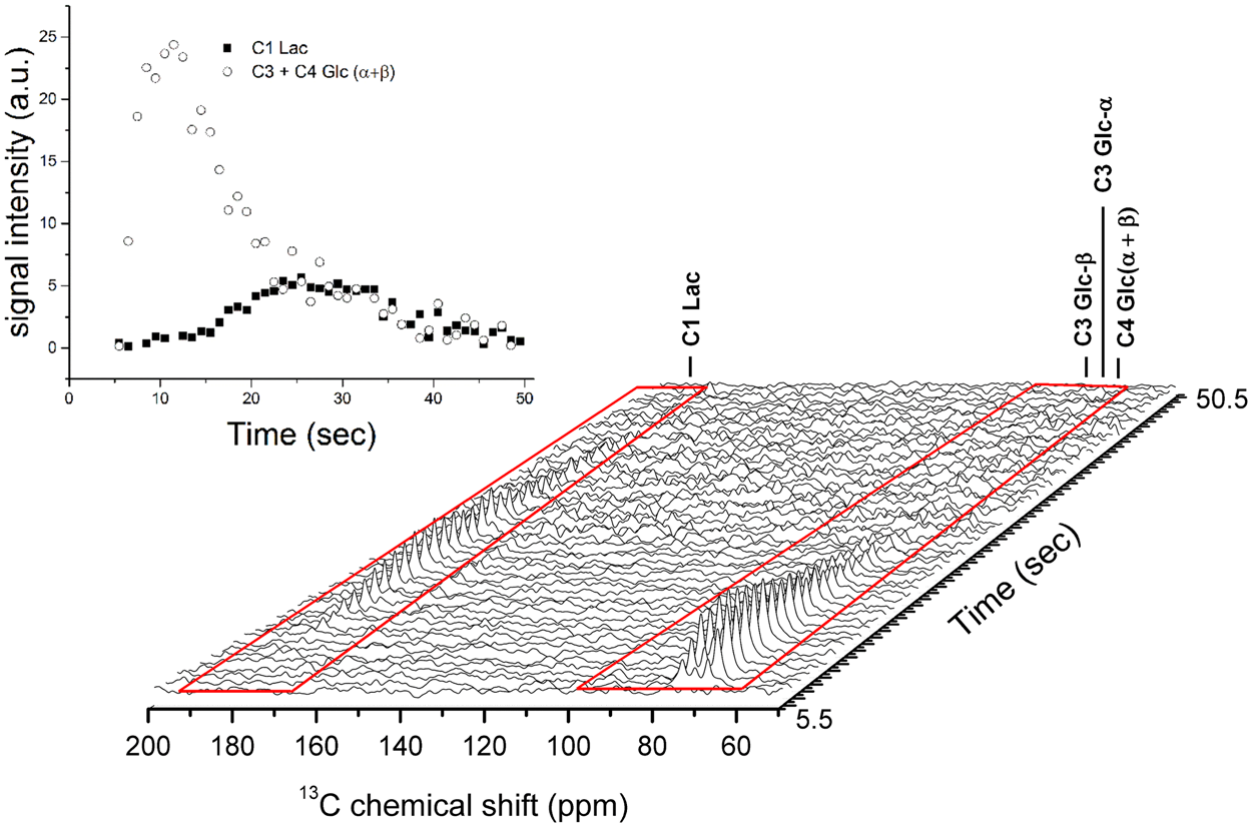
*In vivo* ^13^C MRS in the mouse head following the injection of hyperpolarized [2,3,4,6,6-^2^H_5_, 3,4-^13^C_2_]-D-glucose. Spectra were acquired by applying 20° flip angle at the carboxyl resonances and −0.05° flip angle at the substrate resonances every 0.5 s. To improve the SNR every two spectra were added leading to effective time resolution of 1 s. Glucose peaks appear at 75.3 ppm (C3-β), 73.8 ppm (C3-α) and 70.7 ppm (C4-α and C4-β) and lactate is observed at 183.5 ppm. The corresponding time course (inset) of the spectral data was quantified using AMARES.

When using [2,3,4,6,6-^2^H_5_, 3,4-^13^C_2_]-D-glucose, the doublet peaks detected at the same frequencies of the three glucose metabolites were coalesce into single peaks due to the lack of the ^13^C-^13^C coupling. The peak at 179.8 ppm could then be tentatively assigned to [1-^13^C] 3PG, (see Fig. 4). The high impurity content of the perdeuterated [U-^13^C] glucose used for the present experiments lowered the quality of the spectra and the low SNR of the pyruvate and [1-^13^C] 3PG signals made it difficult to obtain the time evolution of those two metabolites. Thanks to the high SNR of the [1-^13^C] lactate signal, ranging from 4.3 to 21.6, we could however record the kinetics of the lactate formation (Figs 3 and 5). The lactate-to-pyruvate ratio was calculated for each experiment separately by summing the spectra recorded after a single injection of hyperpolarized glucose in each mouse. This ratio was quantified in 5 mice, including 3 injected with perdeuterated [U-^13^C_6_] glucose and 2 injected with [2,3,4,6,6-^2^H_5_, 3,4-^13^C_2_]-D-glucose, and we obtained a mean value of 17.6 ± 2 (n = 5, mean ± SD).

## Discussion

This study reports for the first time that ^13^C-lactate and ^13^C-pyruvate signals can be detected in the mouse brain as rapidly as 10 s following an i.v. injection of hyperpolarized ^13^C-glucose despite its relatively short longitudinal relaxation time. The lactate-to-pyruvate ratio is in agreement with earlier measurements in the mouse brain^28,29^ indicating that after the 50.5 s elapsed from the injection, metabolism had reached steady-state. The blood glucose concentration did not exceed the typical values used in glucose tolerance tests^30^, with a maximum blood concentration of 17 ± 3 mM following injection. The experimental protocol designed for this study includes optimization of sample formulation, combined with rapid dissolution and transfer and efficient MRS acquisition scheme enabled to dynamically measure lactate formation in the brain in real time during 30 s. The excellent SNR of the experiment provides the opportunity to quantify the signal intensities and to define the time evolution of the lactate formation. This kind of data could then be used for modeling to obtain metabolic rates and as an alternative to directly quantify CMRglc by MRS^31^.

An ad-hoc chemical synthesis designed to deuterate and specifically ^13^C-label the glucose carbons 3 and 4 was developed for this study. These correspond to the two ^13^C nuclei labeling the carboxyl group of pyruvate and lactate and they have the longest ^13^C T_1_ in those metabolites. The similar T_1_ values of the 3,4-^13^C_2_-labeled and the uniformly labeled derivatives indicates that ^13^C homonuclear coupling is not a major source of relaxation. Moreover, this synthesis is general and could be used to deuterate any specifically ^13^C-label glucose that could potentially be used as hyperpolarized metabolic probes.

Using specifically ^13^C-labeled glucose, transfer of the ^13^C-enriched carbon from glucose to lactate is less ambiguous and is not influenced by potential confounding pathways such as the pentose phosphate pathway or scrambling of carbons through the various transketolase and transaldolase enzymes. In our case using hyperpolarized [2,3,4,6,6-^2^H_5_, 3,4-^13^C_2_]-D-glucose and with the detection at the carboxyl carbon resonances (160–190 ppm), there is less ambiguity in the peak assignment of the resonance at 179.8 ppm which we have tentatively identified as the glycolytic intermediate 3PG. Further investigations will be necessary to confirm this assignment. Additionally, it led to a substantial improvement in the ^13^C MRS SNR mainly due to the absence of ^13^C-^13^C splitting in the detected metabolic products of [2,3,4,6,6-^2^H_5_, 3,4-^13^C]-D-glucose. It is nevertheless important to mention that perdeuterated [U-^13^C_6_]-D-glucose has also allowed us to determine the lactate-to-pyruvate ratio and to monitor the build-up of lactate, so specific ^13^C labeling of glucose is not mandatory for all *in vivo* studies.

Our findings demonstrate that hyperpolarized ^13^C-glucose might provide complementary kinetic data when compared to ^18^F-FDG PET that provides information on glucose phosphorylation and has been shown to behave differently from glucose shortly after injection^32^. Abnormal glucose metabolism is indeed implicated in several diseases, including neurodegenerative diseases^33^, and the application of the method reported here, perhaps also in combination with thermally-polarized ^13^C-labeled glucose should prove useful in metabolic studies in various animal models. Note that for quantitatively assessing real-time glucose cerebral utilization, it may be necessary to use a different anesthetic since isoflurane is well known to affect cerebral metabolism^34,35^.

## Methods

### Glucose synthesis

[3,4-^13^C_2_]-D-glucose (97–98%) was obtained from Cambridge Isotopes Laboratories (Tewksbury, Massachusetts) and Omicron Biochemicals Inc. (South Bend, Indiana). [U-^2^H_7_, U-^13^C_6_]-D-glucose, deuterium oxide (D, 99.9%) and deuterochloroform (99.8%) were obtained from Cambridge Isotopes Laboratories (Tewksbury, Massachusetts). Ruthenium (10% on activated carbon, reduced) was obtained from Alfa Aesar. Silica gel (60 Å, 65 × 250 mesh) for flash chromatography was obtained from Sorbent Technologies (Norcross, Georgia). The free radical polarizing agent trityl OX063 was obtained from Oxford Instruments Molecular Biotools (Tubney Woods, United Kingdom). Other reagents and solvents were obtained from Sigma-Aldrich (St. Louis, Missouri). All reagents and solvents were used without further purification.

### Methyl [3,4-^13^C_2_]-D-glucopyranoside (1)

Commercially available [3,4-^13^C_2_]-D-glucose (1.00 g, 5.49 mmol) was dissolved in dry methanol (300 mL). Concentrated sulfuric acid (0.500 mL) was added and the solution was stirred at reflux (~80 °C bath temperature) overnight. Upon completion and to avoid side reactions due to the presence of concentrated sulfuric acid, the reaction mixture was treated with freshly prepared Dowex (1 × 4) anion exchange resin in the hydroxide form to remove excess acid. The beads were then filtered and washed with water. The reaction mixture was concentrated by rotary evaporation. The resulting pale-yellow syrup was impregnated with silica gel and subjected to flash chromatography with dichloromethane containing increasing amount of methanol (7.5% to 15%). The fractions containing the product were combined and evaporated to give a clear syrup (1.00 g, 93% yield). The product was a mixture of the α (60%) and the β (40%) anomers as indicated by the NMR data. ^1^H NMR (600 MHz, D_2_O) δ 3.21–3.29 (m, βH2), 3.32–3.39 (m, αH3), 3.41 (s, αCH_3_), 3.43–3.55 (βH3, βH4, βH5, αH2), 3.56 (s, βCH_3_), 3.61–3.65 (br m, αH4, αH5), 3.68–3.79 (αH6_a_, βH6_a_,), 3.85 (br d, J = 12.3 Hz, αH6_b_), 3.91 (br d, J = 12.3 Hz, βH6_b_), 4.36 (d, J = 8.0 Hz, βH1) 4.8 (overlapping with HOD signal, αH1). ^13^C NMR (150 MHz, D_2_O) δ 70.26 (d, J_CC_ = 38.8 Hz, αC4), 70.36 (d, J_CC_ = 39.3 Hz, βC4), 73.80 (d, J_CC_ = 38.5 Hz, αC3), 76.47 (d, J = 39.34 Hz, βC3). HRMS (ESI-TOF, positive mode) *m/z*: [M + H]^+^ calculated for C_5_^13^C_2_H_15_O_6_ 197.0936, found [M + H]^+^: 196.9911.

### Methyl [2,3,4,6,6-^2^H_5_-3,4-^13^C_2_]-D-glucopyranoside (2)

Compound **1** (900 mg, 4.59 mmol) was dissolved in deuterium oxide (~20 mL). 10 mol % ruthenium on carbon (Ru/C) was added to the solution. The reaction vessel was purged with hydrogen gas and equipped with a hydrogen filled balloon. The reaction was heated to 80 °C (bath temperature) and stirred for 24 hours. The reaction mixture was filtered through a Celite S plug and washed with copious amounts of methanol. The filtrate was concentrated by rotary evaporation. If NMR indicated insufficient deuteration, the hydrogen-deuterium exchange reaction was repeated on the crude pale-yellow syrup filtrate. Upon verification of sufficient deuterium incorporation, the pale-yellow syrup was impregnated on silica gel and subjected to flash chromatography with dichloromethane containing increasing amount of methanol (5% to 15%). The fractions containing the product gave a clear syrup (857 mg, 93% yield). The product was a mixture of the α (65%) and the β (35%) anomers as indicated by the NMR data. ^1^H NMR (600 MHz, D_2_O) δ 3.41 (s, αCH_3_), 3.43 (br βH5), 3.56 (s, βCH_3_), 3.62 (br, αH5), 4.36 (br, βH1), 4.79 (br, overlapping with the HOD signal, αH1). ^13^C NMR (150 MHz, D_2_O) δ 69.76 (doublet of triplets J_CC_ = 38.5 Hz, J_DC_ = 22.0 Hz, βC4) 69.87 (doublet of triplets J_CC_ = 38.8 Hz, J_DC_ = 22.2 Hz, αC4), 73.24 (overlapping doublet of triplets J_CC_ = 38.5 Hz, J_DC_ = 22.1 Hz, αC3), 75.89 (overlapping doublet of triplets J_CC_ = 39.1 Hz, J_DC_ = 21.5 Hz, βC3). HRMS (ESI-TOF, positive mode) *m/z*: [M + H]^+^ calculated for C_5_^13^C_2_H_10_D_5_O_6_ 202.1250; Found 202.0314.

### [2,3,4,6,6-^2^H_5_, 3,4-^13^C_2_]-D-glucose (3)

Compound **2** (496 mg, 2.47 mmol) was dissolved in freshly prepared 1 M hydrochloric acid (~30 mL). The reaction was heated to ~100 °C (bath temperature) and stirred for 5 hours. Upon completion, the solution was concentrated by rotary evaporation. The resulting syrup was impregnated with silica gel, and subjected to flash chromatography with dichloromethane containing increasing amount of methanol (7.5% to 20%). The fractions containing the product were combined and evaporated to give a clear syrup (393 mg, 85% yield) that crystallized on standing. The product was a mixture of the α (35%) and the β (65%) anomers as indicated by the NMR data. ^1^H NMR (600 MHz, D_2_O) δ 3.43 (br m, βH5), 3.80 (br m, αH5), 4.62 (s, βH1), 5.21 (d, J = 5.5 Hz, αH1). ^13^C NMR (150 MHz, D_2_O) δ 69.86 (overlapping doublet of triplets J_CC_ = 39.3 Hz, J_CD_ = 22.0 Hz, C4), 69.90 (overlapping doublet of triplets J_CC_ = 38.2 Hz, J_DC_ = 22.0 Hz, C4) 72.98 (doublet of triplets J_CC_ = 38.5 Hz, J_DC_ = 22.0 Hz, αC3), 75.92 (doublet of triplets J_CC_ = 39.1 Hz, J_DC_ = 21.5 Hz, βC3). HRMS (ESI-TOF, positive mode) *m/z*: [M + H]^+^ calculated for C_4_^13^C_2_H_8_D_5_O_6_ 188.1093; Found 188.0218.

### DNP sample preparation

Polarization media was prepared from dissolving trityl radical Ox063 (tris{8-carboxyl-2,2,6,6-benzo(1,2-d:5-d)-bis(1,3)dithiole-4-yl-methyl sodium salt) in de-ionized water to yield concentrations of 35 mM or 40 mM. DNP samples were then prepared by dissolving either [U-^2^H_7_, U-^13^C_6_]glucose or [2,3,4,6,6-^2^H_5_, 3,4-^13^C_2_]-D-glucose in 1.1 part polarization medium resulting in final trityl radical concentrations of 22 and 25 mM, respectively. The glucose concentrations in these samples were approximately 3 M. Note that for this preparation addition of a glassing agent (*e*.*g*. glycerol or DMSO) is not needed since glucose in high concentration acts as a glassing agent itself.

### Dynamic nuclear polarization at 7T/1K

The samples designed for *in vivo* experiments were all polarized at 7 T and and 1 ± 0.05 K using a custom-designed DNP polarizer described in earlier publications^25,36^. The microwave power at the output of the source was set to 50 mW and the irradiation frequency was set to 196.8 GHz. The nuclear polarization was monitored as a function of time by means of pulsed NMR using 5-degree tipping pulses. Following DNP polarization, the hyperpolarized ^13^C-glucose solutions were rapidly dissolved and transferred into an infusion pump placed inside the bore of a 9.4 T imager, with a delay between dissolution and infusion set to 3 s^37^. The pump was programmed to automatically inject 500 µL of the hyperpolarized solution (glucose concentration ~100 mM) into a mouse femoral vein.

### Animal preparation

All experimental procedures involving mice were approved by the regulatory body of the Canton Vaud, Switzerland (Service de la consommation et des affaires vétérinaires) and all experiments were conducted according to Federal and local ethical guidelines. Animals were housed in a 12 h light/dark cycle, with *ad libitum* access to food and water and were fasted 12 h prior to the hyperpolarized ^13^C-glucose injection. The glucose blood level before injection were within normal physiological concentrations^30^ (4.7 ± 1 mM). *In vivo* experiments were performed on C57BL/6 J female mice (20.5 ± 1.5 g). Animals were anesthetized with 1.5% isoflurane in a 30% O_2_/70% N_2_O mixture and a femoral vein was catheterized for glucose injection. The mouse was placed on a holder along with the infusion pump and the femoral vein catheter was connected to the outlet of the pump. The holder was then inserted inside the scanner. A bolus of 500 µl of hyperpolarized solution at 100 mM ^13^C-glucose concentration was injected within 9 s. The sample contained ~100 µM trityl radical. Mouse physiology was monitored and body temperature was maintained throughout the experiment (body temperature between 37–38 °C and respiration rate at 100 min^−1^ by adjustment of the isoflurane dose). All animals were kept under anesthesia for a maximum time of 2 h. Glucose blood levels were measured from the tip of the tail before the holder was inserted inside the scanner and immediately after the completion of the data acquisition. The animals were euthanized at the end of each experiment with an overdose of pentobarbital.

### *In vivo*^13^C MRS measurements

Measurements were carried out on a Varian INOVA spectrometer (Varian, Palo Alto, CA, USA) interfaced to a 31-cm horizontal-bore actively-shielded 9.4T magnet (Magnex Scientific, Abingdon, UK). RF transmission and reception were performed with a custom-designed hybrid probe consisting of a proton quadrature surface coil and a three-loop 10-mm diameter carbon surface coil placed on the top of the mouse head. High order shimming was performed using the FASTESTMAP protocol^38^. RF excitation was performed using 20° selective Gaussian pulses (250 μs/40 kHz bandwidth) centered at 183.5 ppm and applied every 500 ms, with effective tilt angle of 1.4° at 95 ppm and −0.05 at 73 ppm. The acquisition time was set to 200 ms with a spectral bandwidth of 30 kHz. Data was processed using JMRUI^39^ software and OriginPro®.

### Data availability

All data is available from the authors upon reasonable request.

## Electronic supplementary material

Supplementary information
