# Supplementary material for: Measuring glucose cerebral metabolism in the healthy mouse using hyperpolarized 13C magnetic resonance
Source: Sci Rep. 2017 Sep 15;7:11719. doi: 10.1038/s41598-017-12086-z (PMC5601924; doi:10.1038/s41598-017-12086-z)
Supplement: Supplementary file 1 — Supplementary information [file 41598_2017_12086_MOESM1_ESM.doc]

Supplementary information

**Measuring glucose cerebral metabolism in the healthy mouse using hyperpolarized ^13^C magnetic resonance**

Mor Mishkovsky, Brian Anderson, Magnus Karlsson, Mathilde H. Lerche,

A. Dean Sherry, Rolf Gruetter, Zoltan Kovacs^*^, Arnaud Comment*

*Correspondence:

[Zoltan.Kovacs@UTSouthwestern.edu](mailto:Zoltan.Kovacs@UTSouthwestern.edu)

[Arnaud.Comment@ge.com](mailto:arnaud.comment@ge.com)

**Dynamic nuclear polarization at 3.35 T and 1.2 K.** DNP samples contained 28.5 mg of [U-^2^H_7_, U-^13^C_6_]-D-glucose or [2,3,4,6,6-^2^H_5_, 3,4-^13^C_2_]-D-glucose dissolved in 100 µL of glassing matrix (water 90%, DMSO 10%). The combined volume of the sample after dissolution with the glassing matrix was used to calculate the necessary amount (10 mM) of trityl radical (tris{8-carboxyl-2,2,6,6-benzo(1,2-d:5-d)-bis(1,3)dithiole-4-yl-methyl sodium salt). All samples were polarized in a 3.35 T HyperSense commercial polarizer (Oxford Instruments, Tubney Woods, United Kingdom) at 1.2 K using 100 mW microwave at 94.088 GHz for 1 hour. The NMR spectra were acquired on a 9.4 T VNMRS high resolution spectrometer (Agilent, Santa Clara, California) at 298 K. The decay of polarization was monitored using 5 degree flip angle and 5 s repetition time. The liquid state ^13^C NMR signal enhancements (Table S1) were measured by comparing the signal intensities of the hyperpolarized and thermal spectra taking into account the flip angles and number of scans. The T_1_ values were obtained from the fitting of the magnetization decay data taking into consideration the loss of magnetization due to T_1_ decay and RF pulsing as we previously described.^1^

**Table S1.** Liquid state ^13^C NMR signal enhancements and nuclear spin polarization (Pn) values of [2,3,4,6,6-^2^H_5_, 3,4-^13^C_2_]-D-glucose in water at 298 K and 9.4 T.

| Carbon | β C3 | α C3 | (α + β)C4 |
| --- | --- | --- | --- |
| Run 1 | 32096 | 38301 | 35408 |
| Run 2 | 31442 | 33249 | 32262 |
| Run 3 | 33180 | 38763 | 35130 |
| **Average** | **32240** | **36771** | **34266** |
| P_n_ | 26 | 30 | 28 |

**Table S2.** Liquid state ^13^C NMR signal enhancements and nuclear spin polarization (Pn) values of [U-^2^H_7_, U-^13^C_6_]-D-glucose in water at 298 K and 9.4 T.

| Carbon | (α + β) C6 | (α + β) C2,C3 and C5 | α C1 | β C1 |
| --- | --- | --- | --- | --- |
| Run 1 | 27215 | 33030 | 16515 | 39365 |
| Run 2 | 38755 | 46614 | 16515 | 63431 |
| **Average** | **32985** | **39822** | **16515** | **51398** |
| P_n_ | 27 | 32 | 13 | 42 |

**Figure S1.** Hyperpolarized and thermal ^13^C NMR spectra [2,3,4,6,6-^2^H_5_-3,4-^13^C_2_]-D-glucose and [U-^2^H_7_, U-^13^C_6_]-D-glucose water at 298 K and 9.4 T


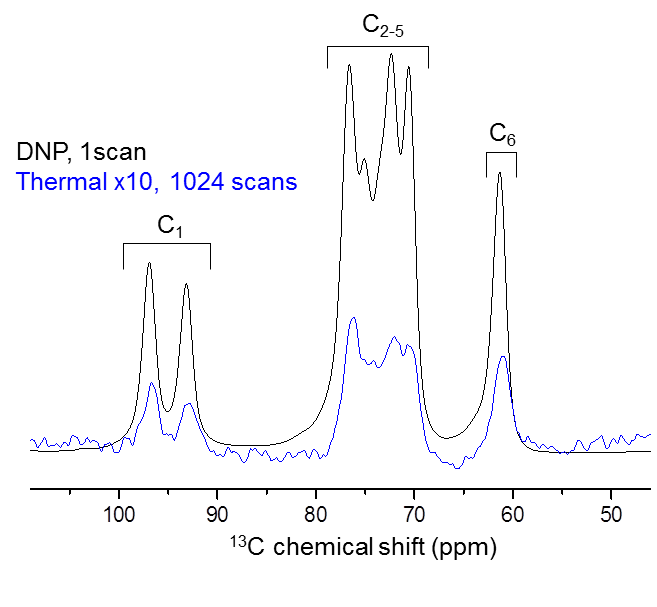


**Figure S2.** Hyperpolarized ^13^C NMR signal of [U-^2^H_7_, U-^13^C_6_]-D-glucose at 9.4 T. The hyperpolarized ^13^C signal was measured inside the injection pump 3s after dissolution and transfer (1 scan, flip angle 5°), and the thermal signal was measured inside the same injection pump overnight (1024 scans with a repetition time of 30 s).

**References:**

1. Lumata, L. L., Jindal, A.K., Merritt, M. E., Malloy, C. R., Sherry, A. D., & Z Kovacs, Z. DNP by Thermal Mixing under Optimized Conditions Yields >60 000-fold Enhancement of 89Y NMR Signal. *J. Am. Chem. Soc.* **133**, 8673-8680 (2011).
